# Women’s and midwives’ views on the optimum process for informed consent for research in a feasibility study involving an intrapartum intervention: a qualitative study

**DOI:** 10.1186/s40814-023-01330-1

**Published:** 2023-06-15

**Authors:** Mary Alvarez, Emily J. Hotton, Sam Harding, Jonathan Ives, Joanna F. Crofts, Julia Wade

**Affiliations:** 1 Department of Women’s and Children’s Health, Southmead Hospital, North Bristol NHS Trust, BS10 5NB Bristol, UK; 2 Bristol Medical School, Bristol University, BS8 2PS Bristol, UK; 3 Translational Health Sciences, Bristol University, Bristol, UK; 4 Research and Innovation, Learning and Research Building, Southmead Hospital, North Bristol NHS Trust, BS10 5NB Bristol, UK; 5 Centre for Ethics in Medicine, Bristol University, Bristol, UK

**Keywords:** Informed consent, Intrapartum intervention, Qualitative research, Maternity, Research ethics

## Abstract

**Background:**

Recruitment to intrapartum research is complex. Women are expected to understand unfamiliar terminology and assess potential harm versus benefit to their baby and themselves, often when an urgent intervention is required. Time pressures of intrapartum interventions are a major challenge for recruitment discussions taking place during labour, with research midwives expected to present, discuss and answer questions whilst maintaining equipoise. However, little is known about these interactions. An integrated qualitative study (IQS) was used to investigate information provision for women invited to participate in the Assist II feasibility study investigating the OdonAssist™—a novel device for use in assisted vaginal birth with an aim to generate a framework of good practice for information provision.

**Methods:**

Transcripts of in-depth interviews with women participants (*n* = 25), with recruiting midwives (*n* = 6) and recruitment discussions between midwives and women (*n* = 21), accepting or declining participation, were coded and interpreted using thematic analysis and content analysis to investigate what was helpful to women and what could be improved.

**Results:**

Recruiting women to intrapartum research is complicated by factors that impact on women’s understanding and decision-making. Three key themes were derived from the data: (i) a woman-centred recruitment process, (ii) optimising the recruitment discussion and (iii) making a decision for two.

**Conclusion:**

Despite evidence from the literature that women would like information provision and the research discussion to take place in the antenatal period, intrapartum studies still vary in the recruitment processes they offer women. Particularly concerning is that some women are given information for the first time whilst in labour, when they are known to feel particularly vulnerable, and contextual factors may influence decision-making; therefore, we propose a framework for good practice for information provision for research involving interventions initiated in the intrapartum period as a woman centred, and acceptable model of recruitment, which addresses the concerns of women and midwives and facilitates fair inclusion into intrapartum trials.

**Trial registration:**

ISRCTN. This qualitative research was undertaken as part of the ASSIST II Trial (trial registration number: ISRCTN38829082. Prospectively registered on 26/06/2019).

**Supplementary Information:**

The online version contains supplementary material available at 10.1186/s40814-023-01330-1.

## Key messages regarding feasibility

- Women were willing to have their recruitment discussions audio-recorded and take part in an interview. The inclusion of vignettes elicited less guarded views on the informed consent process.
- Recruitment to intrapartum trials should be women-centred and acceptable to women. All efforts should be made to inform women of trial information before the birth admission. Most women believed this should be done via the community midwife with whom they have a trusted relationship.
- The findings of the IQS demonstrated that there needed to be alternative approaches and modifications made to the consenting process to facilitate a woman-centred strategy to recruitment, guided by the principles of the framework for good practice (Table 6).

## Background

Informed consent is a not an event, but a complex process [1] central to the ethical conduct of research [2]. Gaining consent for research interventions initiated during the intrapartum period involves added complexities to the norm: not only are women required to assess the potential harm versus benefit to their baby in addition to themselves, but there may also be time pressure for the intervention to be initiated. These complexities add an additional layer of challenge to the usual requirements of consent including understanding the study purpose, the nature of the intervention(s), the right of withdrawal, risks and benefits of participation, how findings will be shared, confidentiality maintained and, in randomised studies, concepts such as equipoise and randomisation. The recruitment discussion, which involves sharing study information and the researcher assessing the woman’s capacity to make a decision to participate, is fundamental to a good quality consent process [3, 4].

During recruitment discussions, recruiters face a dual challenge: conveying a clear account of essential research information [5] and allowing women as much time as they require to be confident with their decision, whilst simultaneously optimising recruitment [6, 7]. Ensuring the potential participant has sufficient understanding to give informed consent can be complicated by factors that are a normal feature of the intrapartum period. At this time, women’s capacity to consent can be affected by, for example, lack of sleep, opiate analgesia and pain [8]. The decision to participate in intrapartum studies is also influenced by contextual factors such as the environment, timing of consent in relation to birth, birthing support, the women’s physical and/or mental state and the accessibility of the study information [9].

In 1997, the Association for Improvements in Maternity Services (AIMS) highlighted the failure of researchers to adequately inform pregnant women of the risks and benefits of participating in research [10]. Inadequate information provision, recognition of vulnerability during labour, inappropriate timing of consent and the lack of women’s involvement in research design led to the mantra ‘research should be undertaken with women, not on women’*.*However, despite this mantra and accompanying recommendations, there is still evidence that this is not always achieved [11].

This qualitative research study investigated women’s and midwives’ perspectives on a model of information provision and informed consent for an intervention initiated in the intrapartum period to identify what was helpful to women and what could be improved, with a view to developing a best practice framework.

## Methods

### Setting

The Assist II study was a non-randomised feasibility study investigating the clinical impact, safety and acceptability of the OdonAssist™ inflatable device for assisted vaginal birth (previously known as the Odon Device) and is an innovative device for use during assisted vaginal birth (AVB—an intrapartum intervention). The OdonAssist™ comprises of a plastic applicator, fastening band and plastic sleeve that slips over the baby’s head—a circumferential air cuff is then inflated and with maternal effort, traction applied to the air cuff to achieve an assisted birth. The mechanism of action and design has been fully described in detail [12]. An integrated qualitative study (IQS) embedded within the larger Assist II study, as a sub-study, investigated the model of information provision and informed consent within the Assist II study and is reported here [12].

The study took place at Southmead Hospital, Bristol—a single tertiary hospital in the southwest of England.

The Assist II study recruitment processes involved distributing the patient information leaflet (PIL) from 20 weeks’ gestation and receiving consent from 28 weeks onwards. Women were approached for a recruitment discussion once in hospital for the birth (spontaneous labour or planned admission). They were offered a PIL, a 10-min study information video and the opportunity to discuss the study with a research midwife. In line with current Royal College of Obstetricians and Gynaecologists (RCOG) guidance for consent to intrapartum research [13], a recruitment approach was permitted from admission to the maternity unit until full dilatation with regional anaesthesia. Research midwives used the ‘hints and tips’ document derived from the Assist sub-study before they began recruitment consultations. It clarified what terminology should be used and avoided and helped communicate the concept of equipoise [14].

### Qualitative study design

Qualitative methods were used to investigate this recruitment process by triangulating findings from the following data sources: in-depth interviews with women invited to take part in Assist II, interviews with Assist II research midwives and audio-recorded Assist II recruitment discussions. The Assist II study [12] trialists were investigating a complex intrapartum intervention. It was decided that integrating a qualitative study into the quantitative stud would allow a more complete analysis and add credibility to the results of the (IQS).

### Participants

IQS participants included a subset of the women invited to participate in the Assist II study [12] and all research midwives involved in study recruitment. Women were eligible to participate in the study if they were anticipating a vaginal birth. Inclusion criteria required women to be 18 years of age and over, have a singleton pregnancy, be over 28 weeks gestation at the time of consent, have a good understanding of the English language, not be in prison, may require an assisted vaginal birth, be at least 36 weeks gestation at the time of the assisted birth, have no latex allergy, have a negative virology, have no intra-uterine death, have no foetal osteogenesis imperfecta, have no foetal bleeding disorders, have no intramuscular or intravenous opiates within 6 h of consent and may consent up to 4 cm of cervical dilatation without regional analgesia or up to full dilatation with regional analgesia.

A purposive sampling strategy was used to include women accepting and declining participation in *Assist II*, with a range of birth experiences (successful and unsuccessful assistance with the OdonAssist™) and a range of characteristics (age, parity and previous birth mode, occupation and ethnicity).

### Data collection

Women participating in the IQS were invited to consent to an audio-recording of the Assist II recruitment discussion and an in-depth interview conducted within 2 weeks of the birth. All six midwives approaching participants within the Assist II study [12] were invited to audio-record all recruitment discussions with potential Assist II participants (with the consent of all involved) and take part in an in-depth interview.

Data collection took place between 3 September 2019 and 4 March 2020. Interviews followed a topic guide informed by existing literature on information provision and consent to interventions in the intrapartum period (Table 1) but allowing interviewees to introduce unanticipated issues of relevance to them.

**Table 1:** Topics covered in in-depth interviews with women and midwives

Women’s interview topic guide explored:	Midwives interview topic guide covered:
• Recollections of the Assist II study • First acknowledgement of Assist II study • The midwives approach • The Assist II information video • The Assist II PIL • The research discussion • Accepting /declining participation • Opinions on diverse research approaches	• Experiences of approaching women • The research discussion and information provision • Challenges of recruiting to an intrapartum intervention • Aiding the decision-making process for women • Conflicting clinical and research roles • Opinions on diverse research approaches

To help elucidate interview participants’ views on the appropriateness of different recruitment strategies, three contrasting vignettes depicting differences in the timing, manner and stage of labour for information provision and approach were used (Table 2). The purpose of the vignettes was to provide scenarios where women and midwives could give opinions on the timing of approaches without necessarily referring to their own experiences.

**Table 2:** The three vignettes used in the in-depth interviews with women and midwives

**Vignette 1—Libby** Libby was admitted to the antenatal ward for an induction of labour at 38 weeks with her first baby. She spent 2 days on the antenatal ward being induced before going to the delivery suite to have her ‘waters broken’ at 3 cm dilated. Whilst in the delivery room, Libby was asked by her midwife if a research midwife could come in and talk to her about a research study called Assist II. Libby agreed, and whilst on the birthing ball having contractions every 4 min, the research midwife gave Libby a leaflet, chatted to her about the study and left her with a video explaining the study in detail. This was the first time that Libby had heard about the Assist II study. Twenty minutes later, the research midwife returned to the room and asked Libby if she would like to take part
**Vignette 2—Cathy** Cathy accompanied by her husband Mike was admitted to the antenatal ward for an induction of labour with her second baby. After being on the ward for an hour she had not yet seen a clinical midwife, but a research midwife came to her bedside and asked her if this was a good time for Cathy to hear about the Assist II study. Cathy was anxious about her induction, but the research midwife was understanding and answered all the clinical questions she had, so she felt able to listen. Cathy was given a leaflet and watched the study video with Mike. The research midwife answered all their questions and Cathy having sought support from Mike agreed that she would participate. She also agreed to her recruitment consultation being audio-recorded and to taking part in an interview with a senior research midwife at home 2 weeks after the birth of her baby
**Vignette 3—Agnes** Agnes was pregnant with her first baby and whilst attending her antenatal clinic at 34 weeks noticed a new poster advertising a research study called the Assist II study. Whilst waiting for her appointment Agnes downloaded the QR code from the poster onto her phone and read a little about the study before she was called to see the midwife. Agnes asked her midwife about the study. She gave Agnes a paper information leaflet and directed her to ring the study office in Southmead where there was someone who would answer any questions. Agnes did not call the office. Six weeks later Agnes was admitted to Southmead in labour at 5 cm. Agnes chatted to the research midwife, watched the study video and gave her consent to participate

The first two vignettes represented common scenarios around timing of the recruitment approach and information provision within the Assist II study [12], and the third was constructed to explore the acceptability of an alternative informed consent strategy.

Recruitment discussions and interviews were recorded using a digital voice recorder and were transcribed verbatim, with identifying data removed.

### Data analysis

Reflexive thematic analysis [14] was used to analyse both interview and recruitment discussion data [15] focussing on participants’ recounted experiences for the former, and the nature and content of the discussion for the latter [16]. Content analysis including simple quantification of content identified the most frequent patterns within recruitment discussion data. Data were hand coded by MA to allow familiarisation before using NVivo12 software to organise codes and develop themes. A proportion (20%) of the recruitment discussions and interviews were co-coded by JW and discrepancies discussed and resolved. These multiple data sets enabled triangulation of data relating to each woman’s experience (woman’s interview, recruitment discussion, midwife interview) as well as cross-case comparison resulting in a deeper understanding [17], and an increased confidence in the findings [18]. Initial themes were discussed between MA, JW, EH, SH and JI and further developed and revised, before a final thematic account was created. Reflexivity was used throughout data analysis and collection as a methodological tool to enhance credibility of the findings [19].

## Results

Twenty-five women and six research midwives were included (Tables 3 and 4 for participant characteristics), and 17 of those women had their Assist II recruitment interviews recorded. Five of those 17 women ultimately declined participation in the Assist II study, and the remaining 12 participated. Six midwives (all those working on the Assist II study) agreed to participate in the interviews. Despite all research midwives agreeing to the audio-recording of recruitment discussions in principle, only three engaged fully with this process, with these three midwives capturing fewer than 10 audio recordings between them.

**Table 3:** Demographics of the women who participated in the in-depth interviews

Women (*n* = 25)	
Accepted or declined participation into Assist II	
Accepted	20
Declined	5
Age (years)	
20–25	4
26–35	13
36–40	8
Birth mode	
Spontaneous vertex	9
Forceps	3
Ventouse	3
Emergency Caesarean section	3
Unsuccessful OdonAssist™ birth/Forceps	2
Successful OdonAssist™ birth	5
Ethnicity	
White British	20
Other White European	2
Asian	2
Latin American	1
Parity *before* index birth	
0	17
1	8
First birth mode of multiparous women	
Forceps	4
Ventouse	1
Spontaneous vertex	3
*n* = Paired audio recording	21
*n* = Interview	25

**Table 4:** Clinical midwives’ characteristics

Research midwives (*n* = 6)	
Experience as a clinical midwife (years)	
< 5 years	2
10–20 years	2
> 20 years	2
Research experience (years)	
< 1 year	2
1–5 years	3
> 5 years	1
Assist experience (number of midwives having previous experience on the Assist trial)	
Yes	4
No	2
Employment during Assist II (number of midwives)	
Clinical and research	4
Research only	2
Recruited to other studies (number of midwives)	
Yes	3
No	3
Full-time equivalent	
0.4	5
0.6	1
Number of Assist II recruitment consultations recorded per midwife	
< 10	3
10–19	0
20–30	3
Number of recruitment consultations per midwife paired with in-depth interviews	
0–2	3
3–7	3

Three themes were developed from the data: (i) a woman-centred recruitment process, (ii) optimising the recruitment discussion and (iii) making a decision for two. Quotes are identified as originating either from women’s interviews (e.g. IP0162A, with A indicating accepting participation, D indicating declining), midwife interviews (e.g. Midwife 01) or recruitment discussions RD (e.g. RDP0162A indicates data from the recruitment discussion of a patient who accepted participation).

### Woman-centred recruitment process

Women’s and midwives’ accounts suggested a range of practices that would keep women and their interests at the centre of the recruitment process, most notably that study information should be freely available to all during pregnancy.

If you don’t inform everybody, how could you possibly know which ones to inform? So, it’s better that the information is wider spread. (IP0162A)

The first time I heard about it was when I was having an antenatal appointment prior to even looking at my birth plan... I read a little bit about it then. I was thinking, oh that’s interesting, I wonder what’s that about. (IP0082A)

Women also suggested community midwives should be the first point of contact with study information and research midwives agreed, aware the rapport women develop with community midwives was hard to replicate during an initial research approach in hospital. Further, whilst there was a clear belief that information should be given throughout pregnancy, many women suggested the third trimester was the optimum timing for receiving information.

It’s worth people giving information leaflets out at [community] midwives’ appointments at like 36 weeks … (IP0302A)

You’d build up trust and rapport with your [community] midwife…but when you meet a new member of staff who’s then giving you information about a study, it’s quite a big ask for them to trust you and agree to take part. (IMidwife01)

It was felt that being signposted to information in advance of labour, through collaboration between community and research midwives, would avoid women receiving information for the first time during birth admission, when there is little time to make a considered judgement on participation. This latter experience was reported by 15 women.

It was sprung on me when I was about to be induced .... I think they should do it beforehand, maybe given time like when they go to their next scan or when they do their birth plan...not spring it when you’re about to go into labour. IP0352A

...having the opportunity to think about things, so perhaps have seen it in the hospital but be able to come back and watch [the patient video] again and have more processing time... I’d have been on a better footing then rather than having all the information straight away and making a decision at the time. (IP0162A)

...if they’ve had the information leaflet at least or they know a bit about the study before they come in... they’ve got that little seed in their head...it’s not such a big deal. They’re ready for it in a way. (IMidwife01)

The third vignette involved ‘Agnes’ (Table 2) who was informed of the study prior to labour and initiated discussion with the research midwife in advanced labour. Most women and midwives favoured this scenario, some citing it as ideal.

It sounds like she’d already decided she wanted to do it, she’d already accessed all the information and she actively asked for a midwife to come and speak to her about it. So, yeah, no problem. (IP0234A)

### Optimising the recruitment discussion

Finding the opportune time for midwives to make an approach and initiate a discussion was challenging given that many women were already anxious due to being admitted with a pregnancy complication or being in labour. Women, including those given information and approached about the study for the first time during labour, did not overtly criticise the timing of their own approach. However, in apparent contradiction to this stance, they expressed concern about the timing of approach in ‘Libby’s’ vignette, approached during labour (Table 2), with comments on vignettes possibly providing less guarded insights into women’s views:

I think you’d have to question, is Libby in a sound state of mind...probably in a significant amount of pain, about to face one of the toughest things in her life that should not be the first time someone hears of the study, let alone is asked to consent for research purposes. (IP0264A)

I think I would have listened, tried to watch the video, tried to read or get someone to read it and then tell me or read it to me but then it would probably have been like, do you know what? Just shush please. [laugh] (Participant IP0308A)

Women reported factors that hindered their ability to make an informed choice during labour including pain, pain relief, tiredness, vulnerability and anxiety, the clinical environment and a lack of privacy. Pain relief was perceived to contribute to diminished capacity and some women relied on their partners to support their decision-making.

.. being approached when I was being induced worked for us... so he was able to kind of like, be there and watch [the patient information video] with me...I was drugged at the time [had been administered codeine]. (IP0086A)

The term ‘vulnerability’ was used by six women. It was used to describe being pregnant, labouring alone, a lack of control and feeling scared.

Well, I think the whole pregnancy thing...it certainly made me feel quite vulnerable because it's not something I'd ever experienced before, and I had no control over... (IP0082A)

I was just so scared, in my head I was terrified that he'd be stillborn...So, I think if someone approached me and started giving me information [prior to hospital] ...I think that would probably scare the life out of me for labour even more than it already was scaring me. (IP0086A)

This highlighted the importance of getting the timing right, but also that women were vulnerable in different ways and at different times, meaning the ‘right timing’ will vary between women and reinforced the need for recruitment to be tailored to individual needs.

The midwives recognised that the hospital environment was not ideal for a research approach and were aware that women were unlikely to expect a first approach for research participation during their admission. The second vignette presented a scenario where the midwife initially offered clinical advice, then introduced the ASSIST II study (Table 2). Most women found this acceptable, but midwives raised concerns about this approach in this context, questioning whether it gave priority to research when clinical needs should be addressed first:

I don’t personally think it matters that she was approached by the research midwife before the clinical one. I don’t see the issue with that. (IP0332A)

...the fact that she hadn’t seen a clinical midwife before the research midwife went in, I’d feel a little bit more uneasy about that because that’s not the reason she is here, to take part in a study, the reason she is here is to be induced. (IMidwife06)

Regardless of the timing, however, allowing time to discuss the study with partners in privacy was valued.

She left us with the [Assist II study video] so we could talk to each other and see, because obviously if she was sat there, you’d kind of feel a bit awkward if [partner] didn’t want to do it or something, but yeah, it was quite nice that she left you... you weren’t feeling pressured or anything. (IP0078A)

I’d just like to approach you both about the Assist II study which we are currently running in this hospital, and I believe that [Partner name], you have had a chance to look at the information leaflet. (RCMidwife04)

The midwives reported that providing engaging, accessible and coherent information was, in their experience, important to women. Some modes of information provision, however, were rated more highly than others. The PIL was intended to raise awareness of the study during pregnancy but was criticised by some women and midwives for its appearance, content and length. Midwives noted that decisions to participate were rarely based on its content*,* and this was supported by some women, who described it as wordy and off-putting:

... it was quite wordy almost put you off wanting to read it because you think, oh like if they were shorter paragraphs or whatever you think, oh I can read this in two minutes, done. (IP0215A)

In contrast, women valued the information video’s visual demonstration of how the OdonAssist™ would be used in practice. Midwives valued the video for the clarity it offered women.

I remember being quite impressed that it felt very much like everything had been thought through so that video gave you all the information you needed. (IP0082A)

So, it was the visual explanation of the bag going over the head and how the baby was then moved down the birth canal and out. I remember seeing that bit... (IP0215A)

Before we launched, I thought it was an additional tool to our chat but now I think it’s an instrumental part...I think probably it’s the seeing the device in operation rather than people talking around it. (IMidwife02)

The recruitment discussion was seen as pivotal in promoting understanding. Women commented positively on the content and manner of information provision, regardless of their decision about participation, with sixteen (thirteen accepters and three decliners) recalling the discussion as being essential for decision-making.

I just bombarded her with questions…So, I’d say her knowledge was quite brilliant, reassuring...it gave me confidence. (IP0332A)

…it is a special time for you, your husband, and the midwife, that relationship, so I think it’s important not to impose too much on that, and the researcher I spoke to didn’t. I think she was very courteous in that way, but yeah, I think if you took up too much of someone’s time on the day, it perhaps takes away a little bit of the special experience and I think that just adds more to the argument about giving the information earlier. (IP0181D)

It is worth noting the final point made by IP0181D, which highlights the risk of a recruitment approach imposing on a special time and supports the idea that an approach prior to labour is preferable.

Combined, women asked 40 different questions during recruitment consultations, (presented in Table 5). Most questions focused on the OdonAssist™ and the baby’s ability to breathe during birth, whilst the fewest focused on research follow-up. Six women asked no questions. One research midwife noted that because of the comprehensive nature of the information video, conversations were often brief.

Most women turn round and they’re like ‘oh, I was going to ask you that question actually about the baby breathing, but it was all covered in the video. (IMidwife04)

**Table 5:** All questions asked in the recruitment consultations

Themes/questions	Participant
Knowledge of the OdonAssist™	
What is the OdonAssist™? Never heard of the OdonAssist™	80D, 81A
Is the OdonAssist™ better than other tools?	77D, 228A, 235A, 255A, 200A, 201A
What does the OdonAssist™ look like	201A
What is the OdonAssist™ made from?	215A
Is the OdonAssist™ recyclable?	215A
The OdonAssist in practice	
How many OdonAssist™ attempts had been made?	234A
What fetal positions are suitable to use the OdonAssist™?	234A, 255A
What does a success rate mean?	284A
Were all the OdonAssist™ attempts successful/success rate?	77D, 86A, 235A
What are the pros and cons of the OdonAssist™?	215A,
Is it more complex procedure?	235A
Why does the OdonAssist™ fail?	200A, 215A, 228A
What if the device fails?	78A, 215A, 235A
Is there less of an infection rate with the OdonAssist™?	284A
When is the OdonAssist™ not used?	235A
Negative side effects/risks of the OdonAssist™	215A, 235A, 255A, 302A
The OdonAssist™ and safety	
Does the OdonAssist™ have a CE mark?	86A
Has there been any harm to the baby or mum?	77D
If other procedures are safe, why has the OdonAssist™ been introduced?	215A
Is the ventouse the size of the baby’s head?	77D
Will my baby suffocate from the cuff/plastic?	77D, 86A, 215A, 228A, 255A
Do OdonAssist™ births have an effect on neonatal hearing?	215A
Are forceps too hard for the baby’s head?	77D
Are there any known neonatal injuries from the OdonAssist™ device?	215A,
Do babies have marks/scratches from the OdonAssist™?	235A
Does the OdonAssist™ device contribute to more perineal tearing?	78A, 80D, 302A
The benefit of an episiotomy over the risk of tearing	80D
Do you have to have an episiotomy with an OdonAssist™ birth?	234A
The OdonAssist™ proceedure/birth	
How long will the procedure take?	77D
Do we wait for a doctor to do the OdonAssist™ birth?	255A
What if my baby is distressed and there is a delay to using another instrument after the OdonAssist™?	235A, 255A,
Can the OdonAssist™ harm the baby?	86A, 201A, 215A,
Is the OdonAssist™ quicker or take longer than using other instruments?	86A
Is the OdonAssist™ an elective procedure?	200A
Are the OdonAssist™ operators specially trained or experienced?	82A
Does an OdonAssist™ birth prevent a water birth?	302A
Further information	
Can I search the video on YouTube?	77D
Study follow-up	235A, 255A
What is asked of women in the follow up?	235A
Do I come to hospital for the follow up or the qualitative interview?	302A
No questions	81A, 162A, 179D, 181D, 249D, 308A

‘Good’ conversations were defined by midwives as exchanges where the midwives’ believed women were optimally informed, either because of evidence of previous access to study information or because women were able to ask probing questions.

Good conversations, definitely the majority of women have had the information before. (IMidwife06)

The people that I’ve approached, had a conversation with and walked away feeling like yeah that was really, really good were the ones where they asked the most questions...you feel that they’ve got all the information and they’ve made the right decision. (IMidwife02)

...if they’ve had the information leaflet at least or they know a bit about the study before they come in... they’ve got that little seed in their head...it’s not such a big deal. They’re ready for it in a way. (IMidwife01)

The physical state of the women, access to prior information, the timing and location of the discussion, and the length of time given to make a decision about participation were most influential in how positively women perceived the discussion, irrespective of whether they accepted or declined participation. At interview, 2 weeks postpartum, there was no consistency in the elements of participation that women could recall, although most acknowledged their lack of knowledge about study detail. This inability to recall was reported by women regardless of whether they gave consent on Central Delivery Suite or in pain. Those who had only partial understanding at the time of their decision appeared to accept this as sufficient and place their trust in the study team:

To be honest with you...sort of understood it a little bit, what would happen and why I would need it. (IP0308A)

Other women demonstrated a clear understanding of what they consented to:

I consented to, if the right person was available at the time and I needed an assisted delivery that I would have one. So, I consented to going along with it to the point of delivery and then sort of re-evaluating whether I definitely still wanted at that point. (IP0235A)

Most women were given a short time by the research midwives in which to consider participation, but some indicated having ample time to consider, as illustrated by the following excerpt from a recruitment discussion:

So, I’m around for another few hours now and then I'm here again tomorrow. If you decide to sign up prior to that ask the [clinical] midwife to give me a shout and I'll come back down. (RCMidwife02)

This woman was followed up the next day. After all her queries had been answered, she felt comfortable with her decision to participate.

RCMidwife02: ‘Would you like some time to think about it you wanted to take part in the study?’

RCP0235A: ‘No. I think we’re happy to participate in it’.

### Making a decision for two

One issue above all others influenced decision making: women were making a decision for two. Any other motivation for participation came with the caveat that there must be no risk to their baby.

...as long as it definitely doesn’t hurt little one that you know, I’m happy to help out with the research. That was my only main priority. (IP0308A)

Midwives suggested that some women were taking some control over their baby’s birth by participating. One midwife recalled a woman, traumatised by her first child’s birth, initially rejecting participation before thinking again after her husband made the point that participating gave her another option during labour.

...she said, ‘oh no, I’m really anxious, this is just too much now’...then she came sobbing back into the office....‘my husband just made the really good point of it gives me another option before having to have another caesarean section’. (IMidwife05)

Women also compared the OdonAssist™ to other methods used for AVB. After engaging with the study information most women believed the OdonAssist™ to be a better option, with the absence of hard surfaces on the device leading women to believe the OdonAssist™ was gentler for both themselves and their baby—which suggests an incomplete understanding of equipoise. Twenty women perceived the OdonAssist™ to be a preferable alternative to forceps and participated with the aim of avoiding a forceps-assisted birth.

...I think anyone who’s either had it or nearly had to use forceps, it’s always been like, ‘oh no, they had to use forceps. (IP0264A)

It just looked a bit better and a little bit safer, for me, ‘because it went over the head...I don’t know, just looked a bit safer for me than the forceps. (IP0081A)

Women declining participation in Assist II gave a range of reasons (including fear of something going wrong, concern for the baby’s wellbeing, resistance from family members). When comparing women who declined with those women who accepted participation, none of the decliners had study information prior to the discussion. Most women who declined participation reported doing so because of late information, explaining they would have considered participation with prior information. Decliners also included proportionally more women with English as a second language who did not have translated information available to them (three of five decliners compared to two in twenty of those accepting).

### Framework for good practice

Key principles derived from the above findings are summarised in Table 6. These principles are proposed as a potential framework for good practice for information provision for research involving interventions initiated in the intra-partum period.

**Table 6:** Framework for good practice for information provision for research involving interventions initiated in the intra-partum period

Framework for good practice for information provision for research involving interventions initiated in the intra-partum period	
A woman-centred recruitment process	• Women prefer study information to be first communicated to them by community midwives • Women want Information provision freely available in pregnancy • Consider pathways into the study that enable recruitment prior to labour • Full information disclosure to be available to all women and not limited based on likelihood of an intervention being required • The timing of the ‘right time’ for information provision will vary between women • Women prefer to be recruited outside of the birth admission
Optimising the recruitment discussion	• Women with a prior awareness of the study are more likely to have an interactive discussion • The discussion is pivotal in facilitating understanding and optimised when the woman makes autonomous decisions with full capacity • Acknowledge the barriers to the ‘right state of mind’ when processing information - Pain - Pain relief and tiredness - Vulnerability and anxiety - Clinical environment and gate keeping - Lack of privacy • Engaging, coherent study information is crucial in promoting understanding • Video demonstration aids understanding
Making a decision for two	• Women need time to research and discuss information with family/friends • Communication barriers impact on the diversity of participants • Translated study information provision should be available for women with English as a second language

## Discussion

This study explored the experiences of women and midwives who were invited to participate in, or recruiting into, the Assist II study—an intrapartum research study in which a recruitment approach could take place between 28 weeks’ gestation and full dilatation [12]. The study identified three key themes that capture features of recruitment practice that are important to women and midwives: a woman-centred recruitment process, optimising the recruitment discussion and making a decision for two. One implication for future research is that the information provision and recruitment process within Assist II was largely acceptable to women, as recruitment targets were met more rapidly than anticipated, and women’s views about processes were predominantly positive. Furthermore, as a result of this, IQS modifications for improvement were identified and implemented, to improve the recruitment experience. For example, 4 months into the 16-month recruitment period, informed by the developing themes of this study, the recruitment process in Assist II was changed so that only potential participants who had prior information about the study could be approached in labour. The three themes described above form the basis for a proposed framework for good practice (Table 6), which aims to place women at the centre of the recruitment process using the following principles: that every woman be provided with the opportunity to participate in research, the promotion of women’s autonomy, acknowledging vulnerability, avoiding professional gatekeeping and supporting the understanding of research information.

There is no current guidance from professional bodies stipulating that women who consent to interventions initiated in the intrapartum period should have information before labour. Current recommendation from the RCOG is that study information should be given based on the likelihood of an intrapartum complication occurring. For example, if the likelihood of the complication is greater than 1:10, women should be given only brief study information in pregnancy; if the complication occurs, women should be given full study information and informed consent given ‘at the time of the emergency’ [13]. This guidance is based on 21-year-old research that sought to establish whether women in pain understood the risks associated with the siting of an epidural and is intended to prevent psychological trauma caused by information about events unlikely to happen [20, 21].

We argue that a research discussion is not comparable to counselling for an epidural. An epidural is a clinical procedure carried out for the benefit of the labouring woman, and the threshold for consent is likely to be lower than that for research. Treatment and research are different, and the consent process required is different. The RCOG guidance aims to spare women the full details of intrapartum research in pregnancy, because of the assumed psychological trauma it may cause [13]. Findings from our study suggest this stance may result in exclusion of potential participants from research and so may be viewed as inappropriately paternalistic and disempowering for women. All women in our study, including those who would have chosen not to access the information due to concerns about it increasing their anxiety, were clear that they believed access to this information should be given ahead of the intrapartum period. This position is in line with that taken by AIMS [10] and is consistent the concept of women-centred care as it gives them control.

Woman-centred care, which simply means care that is individualised and developed in consultation with the affected woman, has long been recognised as a quality marker in maternity services, where the woman’s needs, as defined by the woman herself, are prioritised, and she is given choice, control and continuity of care [22]. This model promotes shared decision-making with the intention of satisfying the woman’s individual wishes and clinical needs. We argue that this model will have implications for research practice and should be extended to the research environment where the needs of women take precedence over the quantitative requirements of the study.

Our findings also confirm previous research and have practical implications for information provision. The third trimester was described by most as optimum timing for information provision, when women turned their attention to labour [23–25]. A prior awareness of the study, described as ‘a seed in their head’, was reported to be conducive to accepting a research midwife’s later approach. Midwives’ felt that prior awareness of the study is an essential ingredient of a ‘good’ recruitment discussion, because women with prior awareness were more engaged and asked more questions [24, 26, 27]. Two women preferred not to have the information prior to admission to hospital as they believed it would have increased their anxieties around labour and birth; however, they accepted that information should be available to women, to engage with as they chose. The women who reported being most satisfied with their decision were the women who were given extended periods to decide, sometimes overnight.

A practical and ethical implication and one of the key factors favouring information provision prior to the intra-partum period was the difficulty reported by women, and observed by midwives, in absorbing information and making participation decisions when not in the ‘right frame of mind’. Of the women recruited in labour, none could recall the information provision or discussion, and of note was that the presence of epidural anaesthesia did not make for a better ‘state of mind’. Rather, pain was replaced by exhaustion and difficulty staying awake. In principle, labouring women cannot be considered to lack capacity to make their own decision simply because they are labouring—but both women and midwives in our study felt that decision-making capacity can be impaired during labour.

Similar findings have been reported elsewhere. The ‘Got-it’ study [23] required an intrapartum consent in the event of a retained placenta (the clinical incidence of a retained placenta being approximately 2% of vaginal births). Both researchers and women considered the study information (both written and verbal), given at the time of the emergency, as straightforward and the giving and receiving of consent as uncomplicated. However, qualitative interviews with women and researchers generated conflicting views on whether *valid* consent had been obtained. The researchers felt they had provided clear, succinct information about participation, whereas women felt that they did not fully absorb the information, particularly the risks of participation, because of their inability to engage with researchers after labour and delivery. They did not feel they gave informed consent and would have preferred information antenatally.

This finding was consistent with the views of acutely unwell women in the QUOTE study, which reported their experiences of recruitment to the Magpie trial [25]. The trial investigated the use of prophylactic anticonvulsants for women with severe pre-eclampsia. A key finding was that women suggested that they should have been given information in the antenatal period, even if there was only a small chance of them being able to participate. Such information sharing had been rejected by the QUOTE researchers in the design phase, as they felt a duty of care to not distress the 98% of women who would be ineligible to participate [25].

Women articulated a sense of vulnerability, which they associated with being pregnant, labouring alone, a lack of control or feeling scared. Research midwives recognised this vulnerability and sought to protect vulnerable women from research activities. However, this translated into gatekeeping, choosing which women were approached and which were not. There is an argument that midwives should not act on these protection motives, but hand ownership of the decision to women entirely, and let them decide when and if they receive a research approach [26, 27]. Not offering an approach removes a women’s right to self-determination which is ethically hard to defend as it insults women’s autonomy and most women want information [28]. However, if we accept the potential for vulnerability and loss of capacity to make that decision during labour, it may well be absolutely appropriate (both ethically and legally) for the research midwife to act as gatekeeper and make a decision in the best interests of the labouring women. However, this dilemma of whether to approach or not could be resolved entirely by ensuring that information is given, and a decision about in-principal participation made, prior to labour.

The study video was valued by all women and the OdonAssist™ demonstration stood out as the section most well-recalled, and the most valued feature. The demonstration enabled women to visualise the OdonAssist™ in use, which appeared to play an important and often decisive role in their decision-making. Midwives welcomed the video as it pre-empted many of the women’s questions and anxieties, suggesting that video can be very useful for a specific purpose [29] and may enhance the quality of consent and be of particular benefit in studies like Assist II [12] where demonstration of a procedure is needed.

Women, and most midwives, agreed with other intrapartum research study participants that the PIL was less useful than other modes of information provision [30, 31]. It could be that the necessity of including research governance text in the pamphlet made it unnecessarily long and negatively impacted on presentation and accessibility. However, it may simply be the case that requiring women to read and understand written information at a time of heightened anxiety and vulnerability, in the context of labour, is simply too demanding. If women were given time to engage with the PIL outside of the birth admission, and not in a state of heightened anxiety, they might be able to read, absorb and discuss it, and might have felt differently about it. Either way, what this suggests is the most appropriate of the mode of information provision is likely to be context and person dependent, and the imperative for potential research participants to consume a written PIL, regardless of context, is misplaced. Most women reported the recruitment discussion was pivotal in their decision-making in terms of helping them understand what was involved [9, 30] and more important than any other mode of information provision [24]. Women were impressed with the research midwives’ knowledge, professionalism and kindness. They emphasised the importance of the research midwives’ interpersonal skills, factors widely reported as influencing recruitment to trials [9, 25, 32–35].

The original model of information provision for Assist II had envisaged community midwives providing information in the third trimester, but many women were presenting to the research team on admission to hospital without prior knowledge of the study. To promote Assist II, we placed the study on Facebook and Twitter, used the Trust website to host up-to-date research study information and placed standing banners with QR codes in the maternity unit, and a PIL (with a QR code) was sent to all women with their 20-week scan appointment. The new recruitment strategy, implemented after presenting interim findings form this study to the Assist II team, involved approaching women attending the Maternity Assessment Unit in person, and giving women the opportunity to view the study information video at home, followed by a short telephone discussion with a research midwife. Research midwives reported that women valued this new approach. An important practical implication from this study is that going forward future research requiring consent during the intrapartum period will require research information to be disseminated to the community midwives without a further burden of work, i.e. a slot in an antenatal class or a short video on current studies that is periodically updated and sent to women via the hospital app.

Training is important in studies where midwives are the primary recruiters. There is currently no prerequisite training (outside of good clinical practice) when midwives move into the role of the research midwife. The Assist II study [12] training consisted of a ‘Hints and Tips’ document derived from the findings of the Assist sub-study [36]. The document was an example of a discussion structure that research midwives could personalise [1] and served as an ‘aide memoire’. The women commented favourably on the midwives’ depth of knowledge, reporting this positively influenced their decision making—suggesting that well trained and knowledgeable research midwives are likely to have a beneficial impact on women’s experience.

A limitation of the IQS was that the data comes from a single study and the findings cannot simplistically be generalised across all intrapartum studies—although many of the findings are consistent with existing literature, suggesting that they are not entirely specific to this study setting. Time and resources were limited, so care was taken in the selection of participants and in the prioritisation of the questions asked in the interviews, in order to maximise the richness of the data [37]. The majority of women had accepted participation in Assist II study, and those declining may have had different experiences which were not adequately captured. Participants were mostly White European and minority groups may have had different views about information provision. During the IQS, it became clear that audio recordings for some women approached were not being captured due to midwives’ reluctance to record these data, and insights from the recruitment consultation data were limited by the relatively small number of recordings collected. However, this study captured a range of views and experiences of women and midwives and was strengthened by triangulation of data from the audio recorded recruitment discussions, and interviews with both women and midwives. Questions emerging from this study, which would benefit from further research, include what capacity do midwives have to deliver the required information at the preferred time point?; what factors might increase participation in (trials like) Assist II?; and what factors might discourage participation by women for whom English is not a first language?

## Conclusion

Understanding the views of women and midwives on the optimum process for informed consent for research that involves procedures initiated in the intrapartum period is crucial if the informed consent process is to be optimised. Currently, limited data guides evidence-based practice [27] and there is no clarity, and questionable guidance, on the timing of information provision and the conditions that need to be met before a woman is asked to decide on participation and provide consent. We propose a framework of good practice for moving the consent process to a more woman centred approach, in which recruitment and information provision begins earlier in the pregnancy.

## Supplementary Information

**Additional file 1.**

## Data Availability

The datasets used and analysed during the current study are available from the corresponding author on reasonable request.

## Abbreviations

AVB: Assisted vaginal birth
IQS: Integrated qualitative study
PIL: Patient information leaflet
AIMS: Association for Improvements in the Maternity Services
RCOG: Royal College of Obstetricians and Gynaecologists
