# Supplementary material for: Women’s and midwives’ views on the optimum process for informed consent for research in a feasibility study involving an intrapartum intervention: a qualitative study
Source: Pilot Feasibility Stud. 2023 Jun 15;9:98. doi: 10.1186/s40814-023-01330-1 (PMC10268483; doi:10.1186/s40814-023-01330-1)
Supplement: Supplementary file 1 — Additional file 1. [file 40814_2023_1330_MOESM1_ESM.docx]

| **Domain 1: Research team and reflexivity** |  |  |
| --- | --- | --- |
| Personal Characteristics |  |  |
| 1. | Interviewer/facilitator | Which author/s conducted the interview or focus group?  Lead author – Mary Alvarez |
| 2. | Credentials | What were the researcher's credentials? *E.g. PhD, MD*  Registered midwife Bsc Hons |
| 3. | Occupation | What was their occupation at the time of the study?  Senior research midwife |
| 4. | Gender | Was the researcher male or female?  Female |
| 5. | Experience and training | What experience or training did the researcher have?  The researcher had training given to her by her lead supervisor who is an experienced qualitative researcher. Also attended a University of Bristol qualitative short course which covered interviewing techniques. |
| Relationship with participants |  |  |
| 6. | Relationship established | Was a relationship established prior to study commencement?  The research midwives were managed by the interviewer.  Most of the women were visited on the postnatal ward and all the women received a telephone call prior to the home visit. |
| 7. | Participant knowledge of the interviewer | What did the participants know about the researcher? e*.g. personal goals, reasons for doing the research*  All the midwives had known the interviewer for many years in both clinical and research roles. |
| 8. | Interviewer characteristics | What characteristics were reported about the interviewer/facilitator? e.g. *Bias, assumptions, reasons and interests in the research topic*  The researcher’s occupation and knowledge of the speciality. The researcher was aware of the dearth of information regarding the research discussions for intrapartum research. Reflexivity was considered in the interviewing and analysis of the data. |
| **Domain 2: study design** |  |  |
| Theoretical framework |  |  |
| 9. | Methodological orientation and Theory | What methodological orientation was stated to underpin the study? *e.g. grounded theory, discourse analysis, ethnography, phenomenology, content analysis*  A qualitative methodology was used and qualitative methods - reflexive thematic analysis and content analysis. |
| Participant selection |  |  |
| 10. | Sampling | How were participants selected? *e.g. purposive, convenience, consecutive, snowball*  The participants were purposively sampled from the women who were eligible to participate in the ASSIST II study. |
| 11. | Method of approach | How were participants approached? e*.g. face-to-face, telephone, mail, email*  The research midwives were approached face to face.  The women were approached face to face by the research midwife carrying out the research discussion. |
| 12. | Sample size | How many participants were in the study?  6 research midwives  25 women |
| 13. | Non-participation | How many people refused to participate or dropped out? Reasons?  Two women initially agreed to the study at the time of the discussion but withdrew their consent at the pre interview telephone call. |
| Setting |  |  |
| 14. | Setting of data collection | Where was the data collected? e*.g. home, clinic, workplace*  Midwives were interviewed in a research office at Southmead hospital.  The women were interviewed in their own homes two weeks following the birth of their babies. |
| 15. | Presence of non-participants | Was anyone else present besides the participants and researchers?  All women had their babies with them. Some women chose to have their partner/mother present. |
| 16. | Description of sample | What are the important characteristics of the sample? *e.g. demographic data, date*  The women need to be eligible to participate in the ASISST study – they were all term and expecting a vaginal birth. |
| Data collection |  |  |
| 17. | Interview guide | Were questions, prompts, guides provided by the authors? Was it pilot tested?  Interview guides and vignettes were provided. |
| 18. | Repeat interviews | Were repeat interviews carried out? If yes, how many?  Each participant gave one interview. |
| 19. | Audio/visual recording | Did the research use audio or visual recording to collect the data?  A Dictaphone was used to collect the data. |
| 20. | Field notes | Were field notes made during and/or after the interview or focus group?  Field notes were collected at each interview. |
| 21. | Duration | What was the duration of the interviews or focus group?  The interviews lasted between 30 -90 minutes |
| 22. | Data saturation | Was data saturation discussed?  No data saturation was not discussed |
| 23. | Transcripts returned | Were transcripts returned to participants for comment and/or correction?  No transcripts were returned to participants for correction. |
| **Domain 3: analysis and findings** |  |  |
| Data analysis |  |  |
| 24. | Number of data coders | How many data coders coded the data?  There were two data coders. |
| 25. | Description of the coding tree | Did authors provide a description of the coding tree?  No |
| 26. | Derivation of themes | Were themes identified in advance or derived from the data?  The themes were developed from the data. |
| 27. | Software | What software, if applicable, was used to manage the data?  NVivo12 |
| 28. | Participant checking | Did participants provide feedback on the findings?  Yes the midwives were aware of the findings the women are being sent the published article as promised. |
| Reporting |  |  |
| 29. | Quotations presented | Were participant quotations presented to illustrate the themes / findings? Was each quotation identified? e*.g. participant number*  Yes the participant quotations were used to illustrate the findings and each quotation is identifiable. |
| 30. | Data and findings consistent | Was there consistency between the data presented and the findings?  Yes |
| 31. | Clarity of major themes | Were major themes clearly presented in the findings?  Yes, the three themes were clearly presented: i) a woman centred recruitment process ii) optimising the recruitment discussion and iii) making a decision for two. |
| 32. | Clarity of minor themes | Is there a description of diverse cases or discussion of minor themes?  Yes, outliers were incorporated into the article. |
